# Corticosteroid-Responsive Pulmonary Toxicity Associated with Fludarabine Monophosphate: A Case Report

**DOI:** 10.5505/tjh.2012.50490

**Published:** 2012-12-05

**Authors:** Milda Rudzianskiene, Rasa Griniute, Elona Juozaityte, Arturas Inciura, Viktoras Rudzianskas, Greta Emilia Kiavialaitis

**Affiliations:** 1 Lithuanian University of Health Sciences Hospital, Kauno Klinikos, Oncology and Hematology Department, Kaunas, Lithuania

**Keywords:** Fludarabine monophosphate, non-Hodgkin lymphoma, Pulmonary toxicity

## Abstract

Fludarabine monophosphate is an effective drug for the treatment of lymphoid malignancies. Myelosuppression, opportunistic infections, and autoimmune hemolytic anemia are the most common side effects of fludarabine. Herein we report a 55-year-old female that presented with fever and dyspnea after completing her third cycle of FMD (fludarabine, mitoxantrone, and dexamethasone) chemotherapy for stage IV non-Hodgkin follicular lymphoma. Chest X-ray revealed bilateral pneumofibrotic changes and chest CT showed bilateral diffuse interstitial changes with fibrotic alterations. No evidence of infectious agents was noted. The patient had a reduced carbon monoxide transfer factor (45%). Her symptoms and radiographic findings resolved following treatment with prednisolone. The literature contains several cases of fludarabine-associated interstitial pulmonary toxicity that responded to steroid therapy. Fludarabine-induced pulmonary toxicity is reversible with cessation of the drug and administration of glucocorticosteroids.

**Conflict of interest:**None declared.

## Abstract (tr)

Fludarabin monofosfat lenfoid malignansilerin tedavisinde etkin bir ilaçtır. Fludarabinin en sık görülen yan etkileri myelosupresyon, fırsatçı enfeksiyonlar ve otoimmün hemolitik anemidir. Burada evre IV non-Hodgkin foliküler lenfoma için FMD (fludarabin, mitoksantron ve deksametazon) kemoterapisinin üçüncü kürünü tamamladıktan sonra ateş ve dispneyle gelen 55 yaşında bir kadını bildiriyoruz. Akciğer röntgeninde bilateral pnömofibrotik değişiklikler varken göğüs BT’de fibrotik değişikliklerle bilateral difüz interstisyel değişiklikler görüldü. Herhangi bir enfeksiyöz ajan bulgusu saptanmadı. Hastanın karbonmonoksit transfer faktörü azalmıştı (%45). Belirtileri ve radyografik bulguları prednizolon tedavisiyle geçti. Literatürde steroid tedavisine cevap veren, fludarabinle ilişkili birkaç interstisyel pulmoner toksisite olgusu vardır. Fludarabin tarafından indüklenen pulmoner toksisite ilacın kesilmesi ve glukokortikosteroidlerin uygulanmasıyla geri çevrilebilir.

## INTRODUCTION

Fludarabine monophosphate—a purine analogue—is an effective drug used as a single agent or in combination with other drugs for the treatment of patients with previously treated or newly diagnosed chronic lymphocytic leukemia, as well as those with low-grade non-Hodgkin lymphoma [1,2,3]. Myelosuppression, opportunistic infections caused by Pneumocystis jiroveci, mycobacteria, Candida spp., and other agents, neurotoxicity, and autoimmune hemolytic anemia are the most common side effects of fludarabine. 

The literature includes several reports of fludarabineassociated interstitial pulmonary toxicity. Pulmonary toxicity due to fludarabine responds well to steroid therapy [4,5,6,7,8]. In some reported cases other drugs were administered concurrently with or prior to fludarabine, and pathological confirmation of pneumonitis was not available [4,5,8]. Herein we describe a case of pulmonary toxicity following fludarabine therapy that responded to steroid treatment.

## CASE

**Figure 1:**
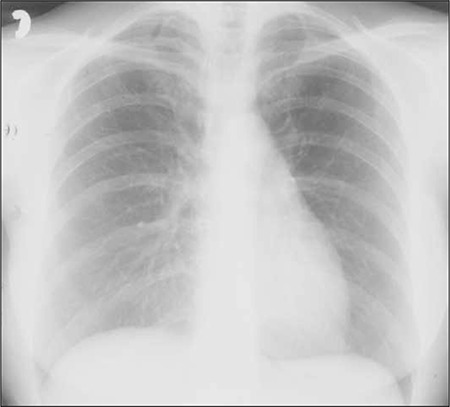
Bilateral pneumofibrotic changes in the chest X ray

**Figure 2:**
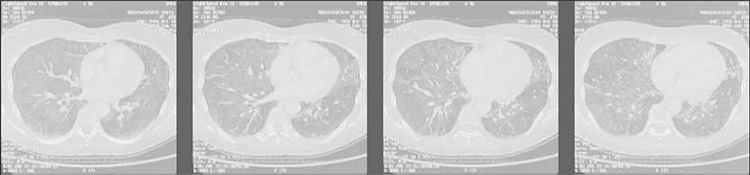
Chest CT: bilateral diffuse interstitial changes with fibrotic alterations in the lower parts of the lungs.

A 55-year-old female with a history of stage IV non- Hodgkin follicular lymphoma was treated with the FMD (fludarabine, mitoxantrone, and dexamethasone) chemotherapy regimen. She was receiving IV fludarabine 25 mg m–2 for 3 d every 28 d. Then, 2 weeks after the last cycle of chemotherapy she was admitted to the hematology department department with fever and dyspnea. Her history of lung disease, exposure to toxins, tuberculosis, and smoking was negative. Her blood pressure was 136/85 mmHg, pulse rate was 116/min , respiratory rate was 36/min, temperature was 38.9 °C, and pulse oximetry was 96% on room air. Examination of the cardiovascular and respiratory systems was normal. Lymphadenopathy and hepatosplenomegaly were not observed. Chest X-ray showed bilateral pneumofibrotic changes (Figure 1); previous chest X-rays were normal. The patient’s laboratory findings were as follows: white blood cell count: 4.7 x 10^9^ L^–1^, with 71% neutrophils, 17% lymphocytes, 11% monocytes, and 1% eosinophils; hemoglobin: 10.7 g dL^–1^; platelet count: 301 x 10^9^ L^–1^; CRP: 47 mg L^–1^. Blood and urine microbiological cultures were negative. Chest CT showed bilateral diffuse interstitial changes with fibrotic alterations in the lower parts of the lungs (Figure 2). Atypical pneumonia or Mycoplasma pneumoniae infection was considered, and empirical treatment with cefuroxime (1.5 g t.i.d. IV) and clarithromycin (500 mg b.i.d. p.o.) was administered for 7 d, but the patient did not respond. 

Clarithromycin was withdrawn after laboratory findings verified that IgM antibodies against M. pneumoniae were negative. Antibodies against cytomegalovirus were also negative. Pneumonitis caused by P. jiroveci was then a consideration and bronchoscopy was performed. The patient was given antibiotic therapy, together with oral trimethoprim/sulfamethoxazole 960 mg t.i.d. for 4 d, but she did not respond. Microbiological cultures of secretions obtained during bronchoscopy were negative for evidence of P. jiroveci. Radiological investigation and bronchoscopy with bronchoalveolar lavage were performed, but no evidence of sarcoidosis was observed. Laboratory data, including ANA, ANCA, anti-DNR, and Scl-70, did not support the existence of systemic rheumatic disease. The reduced carbon monoxide transfer factor (TLCO) was 45%. The findings, including fever, moderate CRP level, diffuse interstitial changes with bilateral fibrotic alterations in the lungs, and a reduced TLCO led to the diagnosis of pneumonitis, and the suspected cause was fludarabine. 

**Figure 3:**
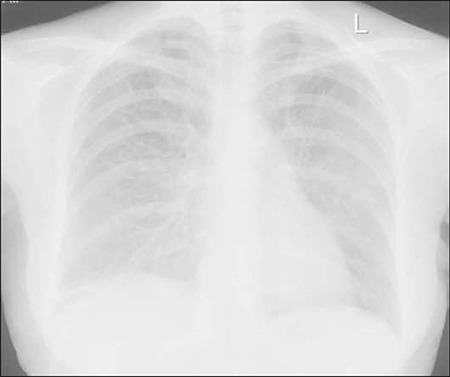
Chest X-ray showed complete resolution of diffuse interstitial changes after prednisolone therapy.

Antibiotic therapy was withdrawn and the patient was started on prednisolone (1 mg·kg^–1^·d^–1^). After 2 d of prednisolone therapy dyspnea and fever disappeared, and chest X-ray showed complete resolution of diffuse interstitial changes (Figure 3). Prednisolone was tapered over the course of 1 month. Post treatment TLCO was 75%. Fludarabine treatment was not reinstated.

## DISCUSSION

Development of diffuse interstitial changes in the lungs can occur due to various reasons, such as connective tissue diseases, drugs, exposure to occupational and environmental toxins, and inherited conditions [9]. Many diverse etiologies of diffuse interstitial changes in the lungs may produce difficulties to establish the cause of pneumonitis. 

Fludarabine monophosphate depletes CD4 cells, altering the CD4:CD8 ratio and producing a syndrome that is clinically and immunologically similar to acquired immunodeficiency [10,11]. The most common infections seen in patients treated with fludarabine are opportunistic infections caused by P. jiroveci, mycobacteria, cytomegalovirus, and Candida spp. [7]. 

Interstitial pneumonitis induced by P. jiroveci presents with acute hypoxia, fever, and non-productive cough. 

Chest X-ray in such cases shows pulmonary infiltrates and a very low arterial oxygen level. Chest X-ray in the presented patient showed diffuse interstitial changes with fibrotic alterations in the lungs, which are not characteristic of P. jioveci infection, and microbiological cultures of secretions obtained from bronchoscopy didn’t show evidence of P. jiroveci, the arterial oxygen level was 96%, and there was no response to trimethoprim/sulfamethoxazole treatment. The observed diffuse interstitial changes strongly suggested atypical pneumonia caused by M. pneumoniae or viral infection; however, viral infection in the lungs was excluded because it does not cause TLCO reduction. Antibodies against cytomegalovirus were negative. Pneumonitis due to M. pneumoniae was excluded because IgM antibodies were negative and there was no response to clarithromycin therapy. Laboratory data did not support the existence of connective tissue diseases. The patient’s anamnesis was negative for exposure to toxins, such as asbestosis, silica dust, and chest radiotherapy. 

The incidence of fludarabine-associated pulmonary toxicity is not known. Helman et al. conducted a retrospective analysis of 105 patients with chronic lymphoproliferative diseases that were treated with fludarabine or fludarabine-containing regimens, and reported an 8.6% incidence of fludarabine-associated pulmonary toxicity [12]. The literature includes several case reports of interstitial pneumonitis related to fludarabine therapy [4,5,6,7,8,13]. In all cases the patients had non-productive cough, dyspnea, and fever, which typically began 1-2 weeks after the last course of chemotherapy. A relationship between the number of chemotherapy cycles and the development of pneumonitis was not established. In the four previous cases patients presented with the certain degree of hypoxia [4,5,8,13]. Garg et al. reported a patient with no signs of hypoxia [7]. In the presented patient pulse oximetry was 96% on room air. Kane et al. reported pneumonitis with severe respiratory failure [5] and Disel et al. reported a case of pneumonitis with rapidly progressing severe dyspnea, cyanosis, and massive pulmonary bleeding [8]. No deaths have been reported. 

Radiographic changes consist mostly of diffuse reticular infiltrates, with or without nodularity, usually in the middle or lower zones of the lungs [7,14]. Chest X-ray in the presented patient showed bilateral pneumofibrotic changes and chest CT showed bilateral diffuse interstitial changes with the fibrotic alterations in the lower parts of the lungs. In all reported cases, as in the presented patient, the causes of infection (viral, bacterial, P. jiroveci, fungal) were excluded and antibiotic therapy was ineffective. In the presented patient a lung function test was performed (TLCO) and the result was 45%. The TLCO is a test used to diagnose, grade, and monitor diseases that affect gas transfer at the alveolar-capillary surface area. The test is useful for identification of disorders that affect lung parenchyma, interstitial lung diseases, and anemia that result from pulmonary hemorrhage, as is seen in chronic thromboembolic disease or pulmonary hypertension [15]. In previously reported cases this test was not performed and interstitial pneumonitis was pathologically confirmed via open lung biopsy in only in 1 published case [5]. 

In previously reported cases, as in the presented patient, corticosteroids were administrated after excluding causes of infection that could have led to the pneumonitis. All reported patients had clinical response and resolution of radiographic findings. Tapering corticosteroid therapy for pneumonitis resulted in relapse in 1 patient, but after steroids were again administered a secondary response was induced [4]. Following reinstatement of fludarabine therapy recurrence of interstitial pneumonitis was observed in some patients [4,6,16]. 

The mechanism of lung injury due to fludarabine is unknown. Response to steroids suggests an immunological mechanism, although direct toxicity cannot be ruled out [7]. Fludarabine therapy can induce pulmonary toxicity, ranging from mild to severe respiratory failure. If symptoms and radiographic findings indicate development of interstitial pneumonitis and possible etiological infections are excluded, fludarabine therapy should be terminated and steroid therapy should be initiated. 

**Conflict of Interest Statement**

The authors of this paper have no conflicts of interest, including specific financial interests, relationships, and/ or affiliations relevant to the subject matter or materials included.
